# Association between serum oxidative stress indicators, inflammatory indicators and suicide attempts in adolescents with major depressive disorder

**DOI:** 10.3389/fpsyt.2025.1539158

**Published:** 2025-02-24

**Authors:** Cao Peng, Mengting Xu, Meng Ye, Weiwei Yang, Maosheng Fang

**Affiliations:** ^1^ Affiliated Wuhan Mental Health Center, Tongji Medical College of Huazhong, University of Science and Technology, Wuhan, China; ^2^ Department of Psychiatry, Fourth People’s Hospital of Nantong City, Nantong, China; ^3^ Department of Psychiatry, Wuhan Mental Health Center, Wuhan, China; ^4^ Shanghai Mental Health Center, Shanghai Jiao Tong University School of Medicine, Shanghai, China

**Keywords:** suicide, MDD, adolescent, oxidative stress, biomarker

## Abstract

**Background:**

Progress in research on the neurobiology of suicide behavior in adolescents has notably lagged compared to that of adults. This study aimed to investigate the associations between serum indicators, including oxidative stress (OS) and inflammatory indicators, and psychological factors with suicide attempts (SA) in adolescents with major depressive disorder (MDD) while also exploring potential markers.

**Methods:**

This study involved the psychological assessment of 106 first-time hospitalized adolescents aged 12 to 18 with MDD and the measurement of serum indicators. Participants were categorized into two groups according to their history of SA within the preceding six months. Screening the best markers for suicide by machine learning algorithms. Multivariable logistic regression was used to assess the correlation between these indicators and suicide. Secondly, Mendelian randomization (MR) was used to initially explore the causal relationship between these serum indicators and suicide.

**Results:**

In adolescents diagnosed with MDD, those who had attempted suicide exhibited elevated serum superoxide dismutase (SOD) activity, reduced nitric oxide (NO) levels, more severe anxiety and depressive symptoms, worse sleep quality, increased exposure to adverse life events, less effective coping strategies, worse parental attachment, more severe alexithymia, and more impulsivity when compared to their counterparts without a history of SA (all p<0.05). The multivariable analyses showed a significant association between serum SOD activity (OR 1.254, 95% CI 1.043-1.534) and anxiety symptoms (OR 1.056, 95% CI 1.020-1.097) with SA in adolescents diagnosed with MDD. The MR analyses showed a causal association between genetically determined low uric acid (UA) levels and a higher risk of SA (OR 0.942 95%CI 0.896-0.991).

**Conclusion:**

Serum SOD activity, anxiety symptoms, and UA levels may be potential markers of SA and suicide intent in adolescents with MDD.

## 1 Introduction

Major Depressive Disorder (MDD) is the predominant cause of worldwide disability, with its start occurring at increasingly younger ages (1, 2). According to the Global Burden of Disease 2019, the prevalence of depression among teenagers aged 10-14 years is 0.98%, whereas it is 2.69% in the 15-19 age group (3). Adolescence is a crucial phase for the anatomical and functional growth of the brain, and episodes of MDD during this era profoundly affect development. Adolescents with MDD exhibit a higher propensity for relapse, experience more severe symptoms, and possess a worse prognosis compared to individuals with adult-onset MDD (4).

Suicide constitutes a substantial global public health issue (5). The World Health Organization (WHO) reports that over 700,000 individuals die by suicide annually (6). Suicide has emerged as the fourth highest cause of death among individuals aged 15 to 29 years (6). Suicide behavior is categorized into subtypes: suicide ideation (SI), suicide attempt (SA), and completed suicide (CS), with adolescence being a critical period for SA and CS (7). Furthermore, approximately one-third of individuals who contemplated suicide and over fifty percent of those who died by suicide were diagnosed with MDD (8). Suicidal conduct is prevalent among individuals with MDD. The one-month prevalence and lifetime prevalence of SA among Chinese patients with MDD are estimated to be 20.3% and 23.7%, respectively (9). Longitudinal research indicates that SA is the strongest predictor of future death by suicide (10). Consequently, early screening and intervention for SA risk in adolescents with MDD is essential for suicide prevention.

Suicide behavior is complex and heterogeneous, with its etiology remaining unclear. Previous studies have identified numerous potential risk factors for suicide. Two cross-sectional studies found an association between sleep disturbances in adolescents and suicidal thoughts and behaviors (STB) (11, 12). Sleep problems can result in STB due to impairments in cognitive, emotional, physical, and social functioning (13). The stress of life events as an environmental factor has also attracted much attention. The integrated motivational-volitional model (IMV) emphasizes the “trigger” role of adverse life events in suicidal conduct (14). A longitudinal study of Chinese adolescents revealed that adverse life events increased the risk of SI (15). Coping abilities are acknowledged as a moderator or mediator between life stress and SI; an absence of coping skills may drive individuals in crisis to pursue extreme solutions (16). A meta-analysis that included 31 studies showed a strong association between lower quality of parental attachment and higher SI in adolescents (17). In addition, personality traits may be important in predicting the long-term risk of SA due to their relative stability (18). Alexithymia is a personality trait that indicates deficits in an individual’s cognitive processing of emotional experiences (19). However, the association between alexithymia and SA is still contentious (20). Moreover, Impulsivity could be an additional personality feature of concern (6). Therefore, these factors were evaluated in this study to obtain more comprehensive results. Nonetheless, these parameters depend on subjective assessments and may be affected by measurement methods, educational attainment, and other variables. Consequently, the development of practical and objective biomarkers is essential for suicide prevention (21).

Oxidative stress (OS) is defined as a disruption of the pro-oxidant/antioxidant equilibrium, resulting in the excessive generation of reactive oxygen species (ROS) or reactive nitrogen species (RNS) (22). Excess ROS/RNS induces oxidative damage to biomolecules and cells, ultimately resulting in many illnesses (23). The brain’s lipid-rich composition, high oxygen demand, and fragile antioxidant barrier make it particularly susceptible to OS imbalances (24). Oxidative damage plays a vital role in the pathophysiology of numerous mental disorders (25). Moreover, evidence indicates that those who have attempted suicide show elevated levels of OS relative to those without a history of SA (26). OS indicators may serve as potential biomarkers for SA. In addition, further research is needed to assess whether there is a causal association between these indicators and SA, which may increase their usefulness as biomarkers for early screening. Randomized controlled trials (RCTs) are the gold standard for establishing causality. However, RCTs are difficult to conduct for various reasons, such as ethical constraints and high costs. Mendelian randomization (MR) is a statistical method that uses Single Nucleotide Polymorphism (SNPs) to simulate RCTs. MR uses genetic variants of the exposure as instrumental variables (IVs) to minimize the effects of confounders and reverse causality. MR can provide reliable estimates of the causal relationship between exposure and outcome under specific assumptions (27).

Investigations into the association between OS and SA in adolescents with MDD are few. This study aimed to investigate the associations between plasma OS indicators, inflammatory indicators, and psychological factors with the risk of SA in adolescents with MDD. In addition, we included suicide intent as a secondary outcome. MR methods were used to assess potential causal associations between these serum indicators and suicide. This research may offer insights for predicting suicide risk in adolescents with MDD.

## 2 Materials and methods

### 2.1 Participants

This is a cross-sectional study of a cohort of adolescents diagnosed with MDD who were hospitalized for the initial time. Data collection occurred during nine months, from March 2023 to December 2023. The inclusion criteria comprised: (a) age between 12-18 years, (b) diagnosed with MDD by two trained psychiatrists in accordance with DSM-5 diagnostic criteria, (c) first-time hospitalized patients with no prior systematic treatment. The exclusion criteria included (a) comorbidity with other psychiatric disorders, such as schizophrenia and pervasive developmental disorder; (b) comorbidity with severe physical illness; (c) comorbidity with physical conditions that impact the immune system, such as infections and chronic inflammatory diseases or the current administration of anti-inflammatory medications.

One hundred twenty people were enrolled in this study. Fourteen individuals were excluded, including six without completing blood tests, three unable to complete psychological assessment, four with infectious diseases, and one using glucocorticoids. Ultimately, 106 patients were included in the study, and none of these subjects were smokers or alcohol drinkers. Each participant underwent a blood draw and psychological assessment within 48 hours of admission. The psychological assessment evaluated the subject’s condition during the past six months. Subjects were classified into the suicide attempt group (“MDD+SA”) if they responded “yes” to the suicide attempt section of the Columbia-Suicide Severity Rating Scale (C-SSRS), while the remaining subjects were classified in the MDD group.

The study was performed in accordance with the Declaration of Helsinki and received approval from the ethics committee of Wuhan Mental Health Center (ethical approval code: KY2023.0310.01). Written informed consent was obtained from all participants and their guardians after they were provided with information about the study protocol.

### 2.2 Methods

#### 2.2.1 Biochemical assessment

Following an overnight fast, approximately 8 ml of fasting venous blood was collected from the participants between 7 a.m. and 9 a.m. the subsequent day. The complete blood counts were obtained using the Automated Hematology Analyzer (BC-6800 Plus; Mindray, Shenzhen, China). The Systemic Immune-Inflammation Index (SII) serves as an objective indicator of the equilibrium between systemic inflammation and immune response status. The SII is computed using the formula: platelet count × neutrophil count/lymphocyte count (28). The remaining blood samples are centrifuged at 3000 rpm for 15 minutes to separate the serum, which is subsequently kept at -80°C until batch assays are conducted. Serum albumin (ALB), total bilirubin (TBIL), and uric acid (UA) were measured using the Bromocresol Green (BCG) method, Diazo method, and Uricase-Peroxidase method on the automatic biochemistry analyzer (LABOSPECT 008AS; Hitachi High-Tech Co., Tokyo, Japan), respectively. Serum glutathione (GSH), superoxide dismutase activity (SOD), glutathione peroxidase activity (GSH-Px), malondialdehyde (MDA), and nitric oxide (NO) were quantified using the DTNB (Ellman’s reagent) method, WST-8 (Water Soluble Tetrazolium-8) method, enzyme catalysis method, TBA (Thiobarbituric acid) method, and nitrate reduction test on the microplate reader (SMR60047; USCN KIT INC., Wuhan, China), respectively. Serum interleukin-6 (IL-6), tumor necrosis factor-alpha (TNF-α), and c-reactive protein (CRP) were quantified using the enzyme-linked immunosorbent assay (ELISA) method on the microplate reader (Type 352; Labsystems Multiskan MS, Finland). Particular assays are detailed in **Supplementary Table 1** of the **Supplementary Material**.

#### 2.2.2 Psychological assessment

Columbia-Suicide Severity Rating Scale (C-SSRS): The C-SSRS consists of several subscales and is a semi-structured clinician-rated interview designed to assess suicide ideation (SI) and behavior (29). The SI severity subscale is a 6-point ordinal scale that ranges from 1 (wish to be dead) to 5 (active SI with specific plan and intent). Adolescents who denied SI were assigned a score of zero. A binary categorical variable was established based on severity scores: with suicide intent (scores of 4-5 points) and without suicide intent (scores of 0-3 points) (30). The SI intensity subscale had five items (frequency, duration, controllability, deterrents, reasons for ideation), each evaluated independently on a 5-point ordinal scale, yielding a total score ranging from 0 to 25. The suicide behavior subscale evaluated behaviors such as suicide attempt (SA), non-suicidal self-injury (NSSI), and suicide preparation.

Zung’s Self-rating Anxiety Scale (SAS) and Zung’s Self-rating Depression Scale (SDS): These two scales assessed participants’ anxiety and depressive symptoms, respectively. Standardized scores for each scale ranged from 25 to 100, with higher scores indicating more severe symptoms. Both scales have shown adequate reliability and validity within the Chinese population (31).

Pittsburgh Sleep Quality Index (PSQI): Participants’ sleep quality was assessed using the Chinese version of the PSQI questionnaire, which has been widely used and validated within the Chinese population (32). The PSQI consists of seven subscales, with higher scores indicating worse sleep quality.

Adolescent Self-rating Life Events Checklist (ASLEC): The ASLEC was used to assess the intensity of adverse life events experienced by participants. The scale consists of six subscales, with higher scores indicating greater stress. The ASLEC has shown good applicability in the Chinese population (33).

Inventory of Parent and Peer Attachment (IPPA): The IPPA assesses the quality of adolescents’ attachments to their parents and peers (34). The IPPA consists of three subscales that assess mother attachment, father attachment (for the father version, replace “mother” with “father” in each question), and peer attachment. Each subscale comprises three dimensions: trust, communication, and alienation (reverse coded). Higher scores indicate a higher quality of attachment. In the study, we computed the average values of mother and father attachment to represent the adolescents’ attachment to their parents. The Chinese version of the IPPA has shown good reliability and validity (35).

Toronto Alexithymia Scale (TAS-26): The TAS consists of four subscales designed to assess the degree of alexithymia. Higher scores indicate higher levels of alexithymia. The scale has exhibited strong reliability and validity (36).

Interpersonal Relationship Integrative Diagnostic Scale (IRIDS): The IRIDS is a tool for assessing interpersonal distress in individuals. It has shown good reliability and validity in the Chinese population (37). The scale comprises four factors, with higher scores indicating more severe interpersonal distress.

Simplified Coping Style Questionnaire (SCSQ): The SCSQ comprises two subscales, positive and negative coping, assessing individual coping patterns. Higher scores on a subscale indicate a greater preference for that coping pattern. The scale has shown high reliability in the Chinese population (38).

Barratt Impulsiveness Scale (BIS-11): The BIS-11 is used to assess the impulsivity of an individual’s behavior. The assessment comprises three subscales, yielding a total score ranging from 30 to 120, with higher scores indicating higher levels of impulsivity. This tool has shown robust psychometric properties in the Chinese population (39).

#### 2.2.3 Statistical analysis

The Kolmogorov-Smirnov test was used to assess the distribution of continuous variables. Continuous variables were presented as mean (standard deviation) if they conformed to a normal distribution; otherwise, they were presented as median (lower quartile, upper quartile). The Independent t-test or Mann-Whitney U test (applicable when variables do not conform to a normal distribution) is used for comparison among groups. Categorical variables were presented as counts (percentages), and between-group differences were analyzed using the chi-square (χ^2^) test or Fisher’s exact test (when expected frequency < 5). Spearman’s correlation test was used to determine the relationship among the metrics within each group of subjects, and the results were visualized by heatmaps. The Benjamini-Hochberg method was used to adjust the p-values in the heatmaps. The least absolute shrinkage and selection operator (LASSO) algorithm and the support vector machine- recursive feature elimination (SVM-RFE) model were used for feature dimensionality reduction. The LASSO algorithm shrinks the variable coefficients by adding a penalty term to achieve feature screening and avoid overfitting. 10-fold cross-validation was used to determine the optimal value of the regularization parameter lambda, and λmin gave a well-performing model with minimal deviance. The SVM-RFE algorithm is based on the maximum margin principle of SVM and eliminates unimportant features by gradual iteration. The characteristics derived by the above two methods were overlaid to identify the best markers for SA. These optimal markers were incorporated into a multivariate logistic regression model to identify independent risk factors. Considering that the severity of depressive symptoms may have a potential impact on suicide risk, we included SDS scores in the model as a sensitivity analysis. Furthermore, independent risk factors for suicide intent were identified using the same methodology. The receiver operating characteristic curve (ROC) was used to assess the effect of these factors on the classification of patients with and without suicide intent, as well as those with and without SA. All statistical analyses and visualizations in this study were conducted using R version 4.3.3 (http://www.R-project.org). A two-sided P value<0.05 was considered significant.

Two-sample MR was used to examine potential causal associations between serum indicators (exposure) and SA (primary outcome). We retrieved genetic data from the Integrative Epidemiology Unit (IEU) open genome-wide association study (GWAS) project (https://gwas.mrcieu.ac.uk/; accessed on 01 February 2025) and the NHGRI-EBI GWAS Catalog (https://www.ebi.ac.uk/gwas/home; accessed on 01 February 2025). The exposure included 10 serum indicators, namely, ALB, TBIL, UA, GSH, SOD, GSH-Px, MDA, CRP, IL-6, and TNF-α. NO and SII were not included in the MR analysis due to a lack of available datasets. The summary data of SA were obtained from two large and independent European ancestry biobank cohorts, the UK Biobank and FinnGen studies (https://r9.finngen.fi/; accessed on 01 February 2025). In addition, SI and suicide behavior were included as secondary outcomes. Detailed information on the GWAS datasets is described in **Supplementary Table 3**. SNPs that met the threshold (p < 5 × 10^-8^, linkage disequilibrium [LD]: r^2^ = 0.001 and clump distance = 10,000 kb) were extracted from the exposure dataset as IVs representing genetic susceptibility. For those traits with IV less than 2 SNPs (including GSH, SOD, GSH-Px, MDA, IL-6, and TNF-α), the relaxed threshold criteria (p < 5 × 10^-6^) were used. The F value for each IV was calculated according to the formula beta^2^/se^2^. IVs with F > 10 were used for subsequent analyses to avoid bias from weak instruments. We then used various statistical methods to estimate potential causal associations between exposure and outcome, including Inverse-Variance Weighting (IVW), MR-Egger, Maximum likelihood, and Weighted Median methods. The MR-Egger intercept test and Cochran’s Q test were used to detect horizontal pleiotropy and heterogeneity, respectively. The random-effects IVW model was used as the primary analysis if significant heterogeneity was present. Otherwise, the fixed-effects IVW model was used as the primary analysis. For SA as the primary outcome, MR analyses were performed in each of two European databases (UK Biobank and the FinnGen study), and these were subsequently meta-analyzed to approximate the average genetically influenced effect on SA. All the MR analyses were performed using the ‘TwoSampleMR’ package (version 0.6.8) in the R software according to the guidelines (https://mrcieu.github.io/TwoSampleMR/).

## 3 Results

This study included 106 adolescents aged 12 to 18 years who were hospitalized for the first time and diagnosed with MDD. **Table 1** summarizes the demographic and clinical features of all participants, with 51 individuals reporting a history of SA in the past six months on the C-SSRS (“MDD+SA group”). There were no significant differences in age, gender, BMI, course of MDD, family history, medication, and NSSI behaviors between the MDD group and MDD+SA group (all p > 0.05). Subjects in the MDD+SA group exhibited significantly higher SI compared to those in the MDD group in terms of both severity and intensity (all p < 0.05).

**Table 1:** Demographic and clinical characteristics of participants.

	Total (n=106)	MDD (n=55)	MDD+SA (n=51)	*p*-value
Age, years^1^	14.0 (13.0, 15.0)	14.0 (13.0, 15.0)	14.0 (14.0, 15.5)	0.134
Female, n(%)	82 (77.36)	40 (72.73)	42 (82.35)	0.342
BMI, kg/m^2^	20.66 (4.07)	20.54 (3.96)	20.78 (4.22)	0.763
Course of MDD, month^1^	12.5 (8.0, 28.0)	12.0 (6.0, 24.0)	13.0 (9.0, 36.0)	0.236
Family history, n(%)	26 (24.53)	12 (21.82)	14 (27.45)	0.654
Medication				
SSRI, n(%)	70 (66.04)	38 (69.09)	32 (62.75)	0.628
Antipsychotic, n(%)	98 (92.45)	53 (96.36)	45 (88.24)	0.224
Mood stabilizer, n(%)	34 (32.08)	15 (27.27)	19 (37.25)	0.372
C-SSRS				
Severity of SI				
continuous^1^	4.0 (2.0, 5.0)	3.0 (0.0, 4.0)	5.0 (4.0, 5.0)	<0.001
by intent, n(%)				<0.001
without intent	47 (44.34)	39 (70.91)	8 (15.69)	
with intent	59 (55.66)	16 (29.09)	43 (84.31)	
Intensity of SI				
composite continuous^1^	12.0 (10.0, 15.0)	11.0 (0.0, 13.0)	14.0 (12.0, 16.5)	<0.001
intensity items
frequency	2.58 (1.62)	1.85 (1.54)	3.35 (1.31)	<0.001
duration	2.17 (1.36)	1.53 (1.21)	2.86 (1.15)	<0.001
controllability	1.86 (1.59)	1.53 (1.27)	2.22 (1.83)	0.026
deterrents	1.55 (1.23)	1.16 (1.08)	1.96 (1.25)	0.001
reasons for ideation	3.10 (1.68)	2.49 (1.94)	3.76 (1.01)	<0.001
NSSI, n(%)	75 (70.75)	36 (65.45)	39 (76.47)	0.302

^1^presented as median (lower quartile, upper quartile)

MDD, major depressive disorder; SA, suicide attempt; BMI, body mass index; SSRI, selective serotonin reuptake inhibitor; C-SSRS, Columbia-suicide severity rating scale; SI, suicide ideation; NSSI, non-suicidal self-injury.

**Table 2** shows the differences in serum biochemical indicators between the two groups. Subjects in the MDD+SA group had higher serum SOD activity (p=0.001) and lower NO levels (p=0.042) compared to those in the MDD group. There was no significant difference between the two groups of subjects in serum inflammatory indicators.

**Table 2:** Comparison of the serum indicators in participants having MDD with and without SA.

	Total (n=106)	MDD (n=55)	MDD+SA (n=51)	*p*-value
OS indicators				
ALB, g/L	45.18 (2.67)	45.37 (2.78)	44.97 (2.55)	0.438
TBIL, umol/L^1^	8.75 (6.62, 11.90)	9.40 (7.20, 13.65)	8.50 (6.30, 10.25)	0.085
UA, umol/L	351.70 (84.26)	355.64 (74.22)	347.45 (94.48)	0.620
GSH, umol/L	74.27 (15.03)	73.93 (12.24)	74.64 (17.67)	0.809
SOD, U/mL	16.87 (2.67)	16.07 (2.24)	17.74 (2.84)	0.001
GSH-Px, U/mL	84.10 (18.22)	81.62 (17.66)	86.77 (18.61)	0.147
MDA, nmol/mL	24.49 (5.26)	24.52 (5.57)	24.45 (4.96)	0.949
NO, μmol/L^1^	12.14 (11.03, 14.90)	13.24 (11.03, 14.90)	11.59 (10.21, 13.79)	0.042
Inflammatory indicators				
IL-6, ng/L	22.48 (1.86)	22.69 (1.85)	22.25 (1.88)	0.231
TNF-α, ng/L	457.04 (37.14)	450.89 (38.71)	463.68 (34.51)	0.076
CRP, μg/L	2494.69 (219.90)	2485.44 (205.98)	2504.66 (235.64)	0.655
SII ^1^	384.31 (269.42, 500.98)	395.76 (241.77, 467.50)	382.01 (287.88, 508.80)	0.395

^1^presented as median (lower quartile, upper quartile)

OS, oxidative stress; ALB, albumin; TBIL, total bilirubin; UA, uric acid; GSH, glutathione; SOD, superoxide dismutase activity; GSH-Px, glutathione peroxidase activity; MDA, malondialdehyde; NO, nitric oxide; IL-6, interleukin-6; TNF-α, tumor necrosis factor alpha; CRP, c-reactive protein; SII, systemic immune-inflammation index.

**Table 3** shows the differences in psychological indicators between the two groups. The results showed significant differences between the MDD+SA and MDD groups in SAS total scores (P<0.001), SDS total scores (P<0.001), PSQI total scores (P=0.001), six dimensions of PSQI except sleeping medications (all P<0.05), ASLEC total scores (P=0.020), interpersonal relationship (P=0.049), punishment (P=0.008), health adaptation (P=0.008), other factors (P=0.011), coping patterns (P=0.003), parent attachment (P<0.001), parent trust (P=0.005), parent communication (P<0.001), parent alienation (P<0.001), TAS total scores (P=0.006), difficulty describing feelings (P=0.004), difficult identifying feelings (P=0.032), interpersonal friendship (P=0.029), BIS total scores (P=0.041), and no planning impulsiveness (P=0.026). Our findings indicate that subjects in the MDD+SA group exhibited more severe anxiety and depressive symptoms, worse sleep quality, more stress, more negative coping patterns, lower attachment to parents, more severe alexithymia, and more impulsivity compared to those in the MDD group.

**Table 3:** Comparison of the psychological indicators in participants having MDD with and without SA.

	Total (n=106)	MDD (n=55)	MDD+SA (n=51)	*p*-value
SAS total scores	55.96 (15.49)	49.42 (14.86)	63.02 (12.94)	<0.001
SDS total scores	68.25 (14.87)	62.93 (15.24)	73.98 (12.21)	<0.001
PSQI				
PSQI total scores	9.94 (4.42)	8.60 (4.04)	11.39 (4.40)	0.001
subjective sleep quality	1.48 (0.85)	1.29 (0.83)	1.69 (0.84)	0.016
sleep latency	1.77 (1.05)	1.49 (1.07)	2.08 (0.96)	0.004
sleep duration	1.08 (1.05)	0.87 (0.98)	1.29 (1.08)	0.038
sleep efficiency	0.71 (1.01)	0.51 (0.84)	0.92 (1.15)	0.036
sleep disturbances	1.50 (0.78)	1.22 (0.66)	1.80 (0.80)	<0.001
sleeping medications	1.26 (1.30)	1.33 (1.29)	1.20 (1.31)	0.605
daytime dysfunction	2.13 (1.01)	1.87 (1.02)	2.41 (0.94)	0.006
ASLEC				
ASLEC total scores	74.43 (15.74)	71.02 (15.21)	78.12 (15.62)	0.020
interpersonal relationship	14.97 (4.20)	14.20 (4.07)	15.80 (4.22)	0.049
learning pressure	14.94 (4.14)	14.76 (4.12)	15.14 (4.20)	0.645
punishment	16.35 (5.13)	15.09 (4.96)	17.71 (5.02)	0.008
health adaptation	11.17 (2.48)	10.56 (2.54)	11.82 (2.26)	0.008
loss	6.82 (3.16)	6.87 (3.44)	6.76 (2.87)	0.862
other factors	10.18 (2.75)	9.53 (2.41)	10.88 (2.94)	0.011
SCSQ				
positive coping	15.99 (7.98)	17.85 (8.71)	13.98 (6.63)	0.012
negative coping	12.58 (5.17)	11.45 (5.18)	13.78 (4.92)	0.020
positive coping pattern, n(%)	25 (23.58)	20 (36.36)	5 (9.80)	0.003
IPPA				
parent attachment	75.51 (17.69)	81.88 (16.73)	68.64 (16.19)	<0.001
parent trust	32.08 (7.86)	34.14 (8.15)	29.87 (6.96)	0.005
parent communication	23.80 (7.32)	26.48 (6.84)	20.90 (6.75)	<0.001
parent alienation	16.37 (4.94)	14.74 (4.10)	18.14 (5.20)	<0.001
peer attachment	77.00 (17.87)	76.91 (18.52)	77.10 (17.34)	0.957
peer trust	31.17 (8.55)	30.85 (8.67)	31.51 (8.49)	0.695
peer communication	23.81 (7.60)	23.51 (7.28)	24.14 (7.99)	0.673
peer alienation	19.98 (5.10)	19.45 (4.59)	20.55 (5.58)	0.271
TAS				
TAS total scores	79.41 (11.68)	76.45 (11.05)	82.59 (11.59)	0.006
difficulty describing feelings	22.49 (4.87)	21.20 (5.29)	23.88 (3.98)	0.004
difficulty identifying feelings	25.25 (5.33)	24.18 (5.35)	26.39 (5.12)	0.032
reduced daydreaming	11.26 (3.84)	11.56 (4.00)	10.94 (3.67)	0.407
externally oriented thinking	20.41 (5.08)	19.51 (5.16)	21.37 (4.85)	0.059
IRIDS				
IRIDS total scores	16.77 (5.63)	15.87 (5.79)	17.75 (5.35)	0.087
interpersonal conversation	5.05 (1.87)	4.75 (1.95)	5.37 (1.74)	0.084
interpersonal friendship	5.37 (1.63)	5.04 (1.64)	5.73 (1.55)	0.029
interpersonal interactions	3.50 (1.84)	3.33 (1.77)	3.69 (1.91)	0.319
heterosexual interactions	2.86 (1.84)	2.76 (2.04)	2.96 (1.61)	0.584
BIS				
BIS total scores	82.26 (14.33)	79.53 (13.97)	85.22 (14.26)	0.041
motor impulsiveness	27.58 (7.54)	27.98 (7.51)	27.16 (7.62)	0.576
cognitive impulsiveness	29.49 (7.66)	28.13 (7.24)	30.96 (7.89)	0.057
no planning impulsiveness	25.19 (8.57)	23.42 (8.50)	27.10 (8.31)	0.026

SAS, self-rating anxiety scale; SDS, self-rating depression scale; PSQI, Pittsburgh sleep quality index; ASLEC, adolescent self-rating life events checklist; IPPA, inventory of parent and peer attachment; TAS, Toronto alexithymia scale; IRIDS, interpersonal relationship integrative diagnostic scale; SCSQ, simplified coping style questionnaire; BIS, Barratt impulsiveness scale.

**Supplementary Figure 1** shows the results of the correlation analysis of various indicators within each group using Spearman’s correlation heatmaps. In the MDD group, 82 correlations were identified, while the MDD+SA group exhibited just 50 correlations. In addition, the results of the heatmap and scatterplot revealed a negative correlation between serum SOD activity and NO levels in the MDD group (*R* = -0.402, *P* =0.002), whereas no such correlation was observed in the MDD+SA group (*R* = 0.111, *P* =0.437). Please refer to **Supplementary Figures 2A–C**. The results of the partial correlation analysis controlling for SDS scores indicated that the negative correlation between serum SOD activity and NO levels was still present in the MDD group (R=-0.431 P=0.001) but not observed in the MDD+SA group (R=0.067 P=0.641). Please refer to **Supplementary Figures 2D–F**.

**Figure 1:**
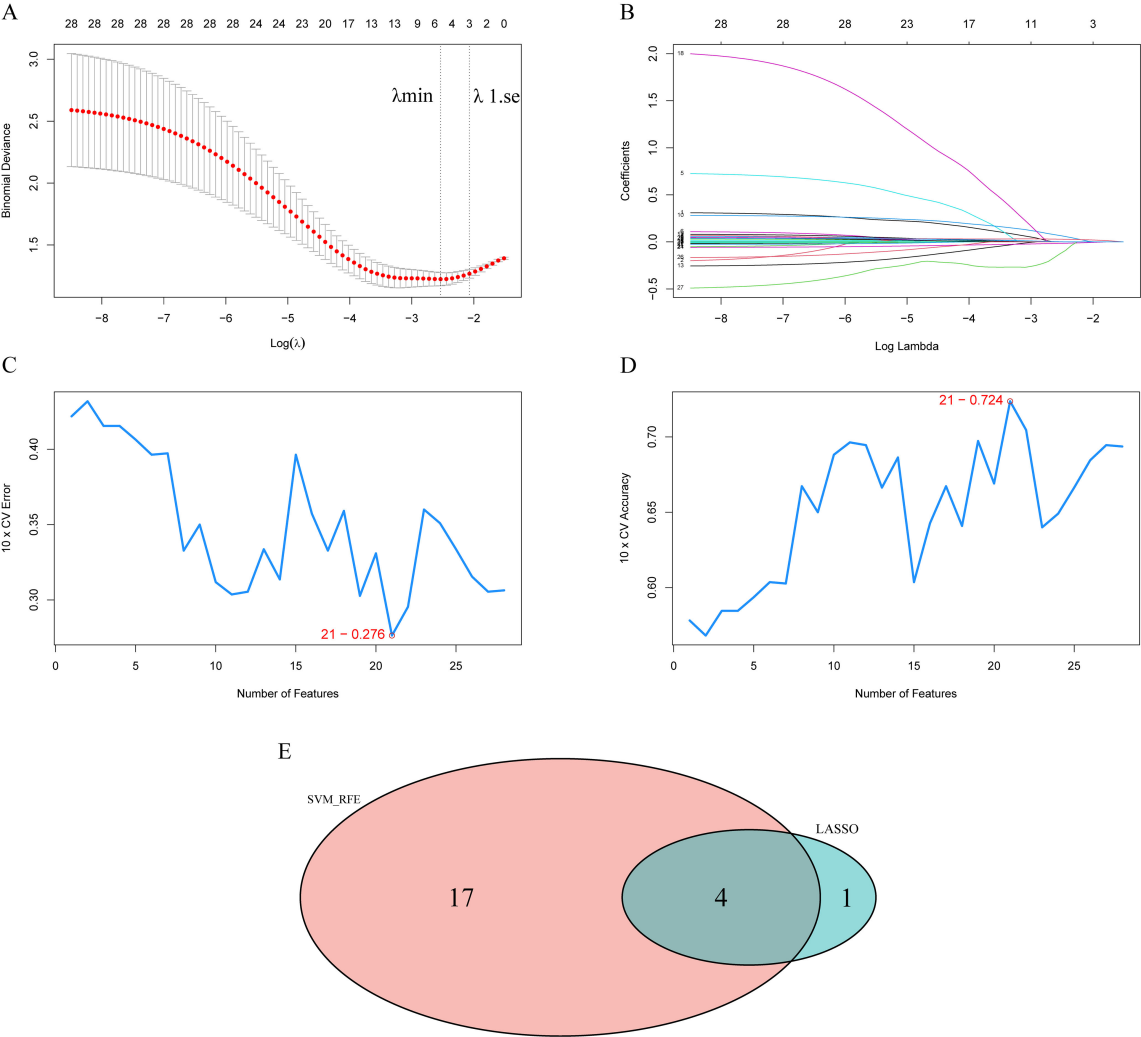
Selection of risk variables using the least absolute shrinkage and selection operator (LASSO) and support vector machine-recursive feature elimination (SVM-RFE) model. **(A)** The variation characteristics of the coefficient of variables; **(B)** the selection process of the optimum value of the parameter λ in the LASSO regression model by cross-validation method; **(C, D)** SVM–RFE model identified 21 candidates with an error of 0.276 and an accuracy of 0.724; **(E)** Venn plot shows the overlapped candidates.

**Supplementary Table 2** shows the results of univariate logistic regression analysis between all indicators and SA risk and suicide intent. To further obtain markers for SA, we screened all indicators by two machine learning methods. By cross-validation, the LASSO logistic regression algorithm identified five indicators, and the SVM-RFE model identified 21 indicators (**Figure 1**). The results obtained by both algorithms were crossed, and finally, four indicators (SOD, SAS, parent attachment, and BIS) were identified as common markers. The results of the multivariate logistic regression analysis suggested high serum SOD activity (OR 1.254, 95% CI 1.043-1.534), total SAS score (OR 1.056, 95% CI 1.020-1.097), and low parental attachment (OR 0.961, 95% CI 0.930-0.990) were independent risk factors for SA. The sensitivity analysis showed that the three independent risk factors remained significant after controlling for the total SDS score (**Table 4**). In addition, we used the same methodology to examine the association between all indicators and suicide intent. Eventually, two indicators (SOD, SAS) were identified as common markers (**Supplementary Figure 3**). The results of multivariate analysis showed that high serum SOD activity (OR 1.259, 95% CI 1.043-1.548) and total SAS score (OR 1.094, 95% CI 1.055-1.142) were independent risk factors for suicide intent and remained significant in the sensitivity analysis (**Table 4**).

**Table 4:** Multivariable logistic regression analysis for identified independent risk factors and sensitivity analysis.

Outcome	Suicide attempt (SA)		Suicide intent	
	OR (95% CI)	*p*-value	OR (95% CI)	*p*-value
Identified characteristics				
SOD, U/mL	1.254 (1.043, 1.534)	0.020*	1.259 (1.043, 1.548)	0.020*
SAS	1.056 (1.020, 1.097)	0.003**	1.094 (1.055, 1.142)	<0.001***
parent attachment	0.961 (0.930, 0.990)	0.010*		
BIS	1.031 (0.999, 1.067)	0.068		
Sensitivity analysis				
SDS	1.000 (0.948, 1.054)	0.998	1.018 (0.969, 1.071)	0.473
SOD, U/mL	1.254 (1.034, 1.550)	0.027*	1.236 (1.016, 1.529)	0.040*
SAS	1.056 (1.009, 1.112)	0.027*	1.082 (1.035, 1.141)	0.001**
parent attachment	0.961 (0.930, 0.990)	0.011*		
BIS	1.031 (0.998, 1.068)	0.071		

*P < 0.05, **P < 0.01, ***P < 0.001.

**Figure 2:**
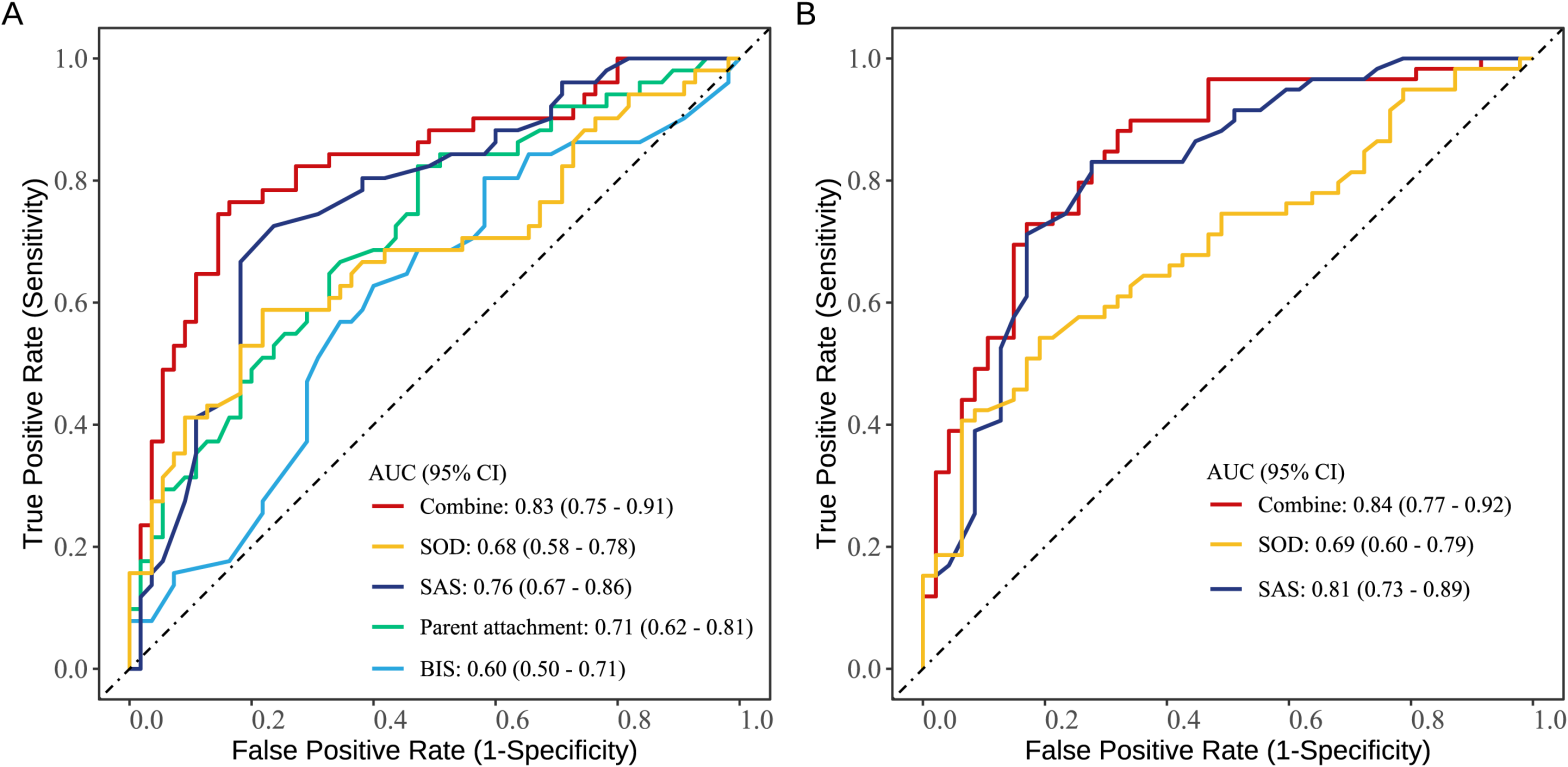
ROC curves of factors identified by multivariable analysis in individually and jointly predicting suicide attempt **(A)** and suicide intent **(B)** in adolescents with MDD.

**Figure 2** presents the results of the ROC analysis. The area under the curve (AUC) of the indicators associated with SA risk are as follows: SOD (0.68), SAS (0.76), parent attachment (0.71), and BIS (0.60). The AUC for the combined prediction of these four indicators was 0.83. The AUC of the indicators related to suicide intent are SOD (0.69) and SAS (0.81). The AUC for the combined prediction of these three indicators was 0.84.

**Figure 3:**
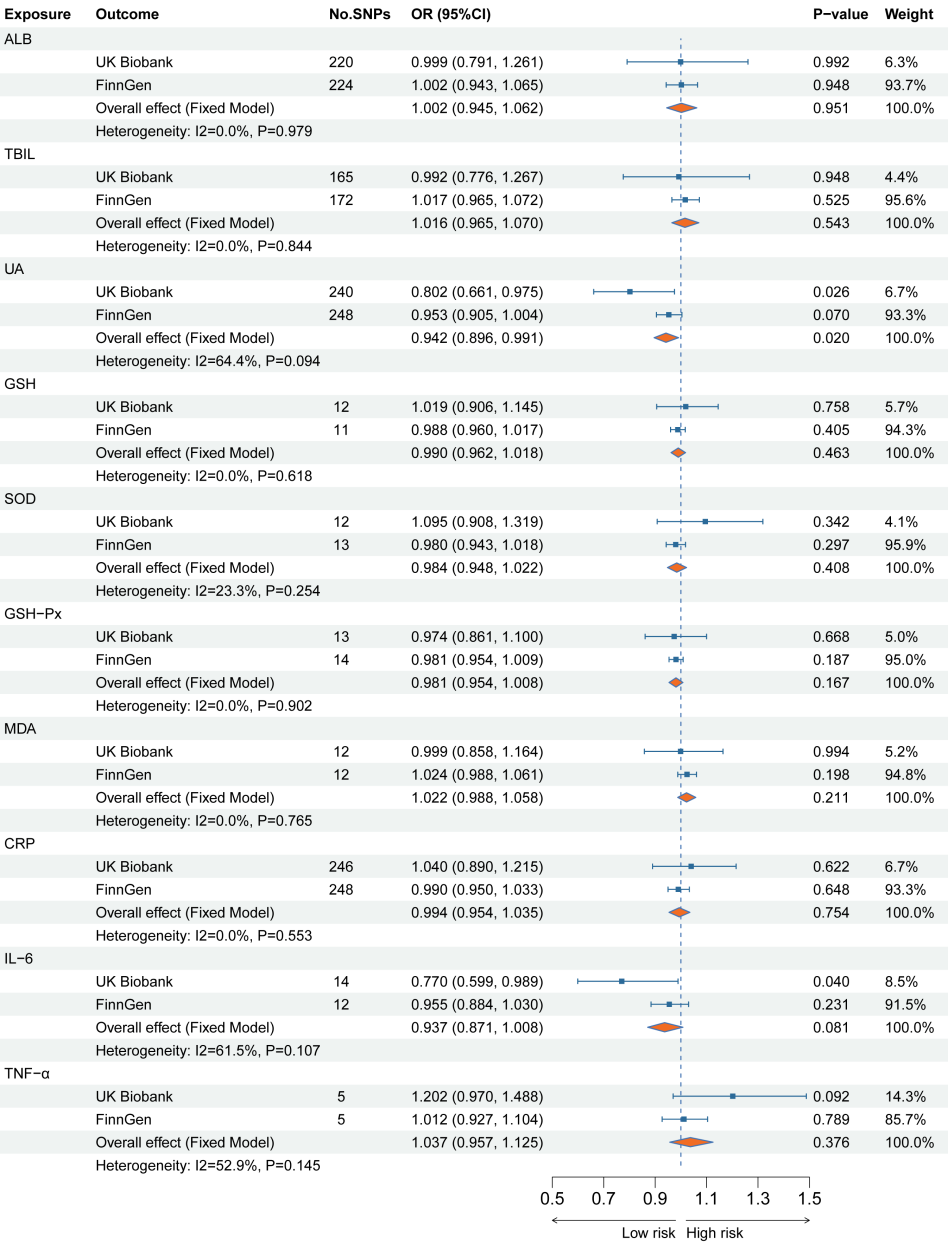
The main MR analysis results for a causal relation of the serum indicator levels on suicide attempts in two independent European cohorts.

**Supplementary Table 4** shows the results of the MR analyses between the ten plasma indicators with SA, SI, and suicide behavior. The MR-Egger intercept test found no significant horizontal pleiotropy for all results. Therefore, the IVW method was used as the main result. **Figure 3** shows the main results of the causal relationship between 10 plasma indicators and SA. The IVW method shows that genetically determined low UA levels were causally associated with an elevated risk of SA in the UKB cohort (OR 0.802 95%CI 0.661-0.975), which is consistent with the results of the Maximum likelihood method (OR 0.802 95%CI 0.665-0.968). Notably, the combined estimates showed that there was still a significant causal relationship between UA levels and the risk of SA (OR 0.942 95%CI 0.896-0.991). In addition, genetically determined low IL-6 levels were causally associated with an elevated risk of SA in the UKB cohort (OR 0.770 95%CI 0.599-0.989), but the combined estimates found no significant correlation between IL-6 levels and the risk of SA (OR 0.937 95%CI 0.871-1.008). No significant associations have been found between genetically determined serum indicator levels and the risk of SI and suicide behavior as secondary outcomes.

## 4 Discussion

In this study, we examined serum OS indicators, inflammatory markers, and psychological factors in 106 adolescents with MDD, comprising 51 with a history of SA and 55 without. Our sample may represent the characteristics of adolescents with MDD who were first hospitalized in psychiatric hospitals in first-tier cities in China. In addition, we used MR to estimate causal associations between genetically determined plasma OS and inflammatory indicator levels and suicide risk.

Our findings indicate that among adolescents with MDD, suicide attempters exhibit more severe anxiety and depressive symptoms compared to those without a history of SA. However, feature screening and multivariate logistic regression analyses revealed that only the SAS total score, which measures the severity of anxiety symptoms, was an independent risk factor for SA and suicide intent. This aligns with previous studies suggesting that anxiety may play a more important role in the suicide behavior of adolescents (40). A longitudinal study (n= 1,265, follow-up 25 years) found that anxiety disorders increased the risk of suicide behavior by 2.8 times, even when controlling for other psychiatric disorders (41). Anxiety has a unique contribution to suicide risk. In addition, our study showed that compared to the MDD group, adolescents in the MDD+SA group had a lower level of trust and communication with their parents and greater feelings of alienation, which is considered to be an insecure attachment relationship. The results of multivariate analyses showed that low-quality parental attachment was an important independent risk factor for SA. According to attachment theory, the quality of the relationship with parents as an early relationship can profoundly influence an individual’s psychological development and emotional well-being (42).

Another finding of this study was that subjects with SA exhibited elevated serum SOD activity and reduced NO levels compared to those without a history of SA. Moreover, elevated serum SOD activity was an independent risk factor for SA and suicide intent in adolescents with MDD. SOD is a powerful scavenger of superoxide anion radicals (O_2_^•-^) and is crucial in combating OS and preventing oxidative damage (43). Previous studies have found elevated SOD activity in both serum (44) and erythrocytes (45) of patients with MDD. Furthermore, two postmortem studies reported elevated SOD activity in the anterior pituitary region (46) and the frontal cortex (47) of suicide completers. This is consistent with our study. The elevated plasma SOD activity in suicide attempters among adolescents with MDD may indicate higher levels of OS in their bodies. The association between NO and MDD, as well as suicide behavior, is still contentious. Some cross-sectional evidence indicates that plasma NO levels and nitric oxide synthase (NOS) activity are diminished in individuals with depression (48–50). A postmortem study revealed diminished NO levels in the anterior pituitary region of suicide completers (46). However, another cross-sectional study found that elevated plasma NO levels were associated with SA in individuals with depression (51). Kim et al. speculated that stress may affect plasma NO levels in individuals with depression bi-directionally by stimulating the NO system and the hypothalamic-pituitary-adrenal (HPA) axis (51). The association between the HPA axis and its critical secretion, cortisol, with suicide behavior has received much attention. Previous studies indicate that dysfunction, particularly hyperactivity of the HPA axis, which regulates the stress response, is associated with suicide risk, regardless of the presence of other psychiatric disorders (52–54). Several studies using the dexamethasone-suppression test (DST) have shown that hypercortisolism is a predictor of suicide behavior in individuals with MDD (52). A recent cross-sectional study also found elevated plasma cortisol levels in suicide attempters (55). Elevated levels of glucocorticoids (GCs) resulting from an overactive HPA axis can reduce NOS expression, resulting in decreased plasma NO levels (51). Notably, this evidence is based on the general population rather than adolescents. The HPA axis in adolescents is immature and more susceptible. Consequently, we recommend that attention to the HPA axis could be enhanced in subsequent studies on adolescent suicide.

We found a negative correlation between serum SOD activity and NO levels in all participants as well as those in the MDD group. However, this relationship was not observed in those in the MDD+SA group. Previous studies reveal a broad interaction between the HPA axis and OS. ROS attenuate the negative feedback system of GCs, while elevated levels of GCs may promote ROS production (56). Increased SOD activity may indicate the presence of high levels of OS and excess ROS. ROS attenuate the negative feedback system of GCs to increase the level of GCs further. The high level of GCs led to a drop in NO levels by decreasing NOS expression. This may explain the negative correlation observed in the study. However, prolonged exposure to stress may lead to attenuation and hyporesponsiveness of the HPA axis (57). Several previous research, including a longitudinal study, have found that individuals with suicide behaviors exhibit blunted HPA axis activity (58–61). Hypoactivity of the HPA axis hinders significant reduction of NO levels in the face of excess ROS. We hypothesize that the HPA axis of some subjects with SA may have become hyporeactive after prolonged hyperactivity, which makes the negative correlation between plasma SOD activity and NO levels disappear.

By MR analysis, we found genetic evidence supporting the causal relationship between UA and SA. Previous studies on this association are controversial. A retrospective study of Chinese adolescent MDD patients (n=533) found an inverse association between UA levels and SA (62), which is consistent with our study. However, another cross-sectional study based on Chinese adult MDD patients (n=271) found no difference in serum uric acid levels between suicide attempters and non-suicide attempters (63). The difference in results may be explained by the different ages of the study populations. In addition, traditional observational studies have difficulty estimating causality, limited by the interference of potential confounders. MR methods minimize the effects of confounders and reverse causation. We performed separate analyses in both databases, followed by a meta-analysis, which increased the robustness of the results. UA is a powerful non-enzymatic antioxidant that scavenges ROS and prevents the oxidation of ascorbic acid, another non-enzymatic antioxidant. Low levels of UA may reflect lower levels of antioxidant defenses and higher levels of OS damage in suicide attempters. All data in the MR analyses were from European populations, avoiding the population heterogeneity bias. However, due to potential inter-ethnic genetic differences, caution is needed when generalizing the MR analysis results to other populations. Although an association between IL-6 and the risk of SA was found in the UK Biobank database, no significant causal relationship was found between the two in the combined estimates. In addition, a less stringent cut-off value (P < 5 × 10^-6^) was used in the process of identifying instrumental variables for IL-6, and the number of SNPs obtained as instrumental variables was still small (No. SNPs = 14). Therefore, this association was considered an opportunity finding.

In conclusion, serum SOD activity and anxiety symptoms may represent potential markers for SA and suicide intent in adolescents with MDD. In addition, there is a causal relationship between genetically determined UA levels and SA. We suggest that serum SOD activity, UA levels, and anxiety symptoms should be assessed more frequently in adolescents with MDD to screen for those at high risk of suicide. However, this finding is still preliminary and needs to be replicated in large-scale studies.

This study has some limitations: (a) Based on published GWAS data, we were unable to test for non-linear causal relationships between these serum indicators and suicide. In addition, some serum indicators have limited IVs, which may lead to false negatives. (b) The general population was not used as a control group in this study because the primary goal of this study was to explore the characteristics and possible biomarkers of suicide attempters among adolescents with MDD. (c) The proportion of male participants in this study was relatively small. However, previous studies have found that females are twice as likely as males to report a history of SA and are more likely to make a first attempt earlier in life (7). This suggests that our sample may be more in line with clinical reality. In addition, the absence of smokers and alcohol drinkers in our sample avoids the potential effects of tobacco exposure and alcohol exposure.

## Data Availability

The raw data supporting the conclusions of this article will be made available by the authors, without undue reservation.
