# Supplementary material for: Association between serum oxidative stress indicators, inflammatory indicators and suicide attempts in adolescents with major depressive disorder
Source: Front Psychiatry. 2025 Feb 24;16:1539158. doi: 10.3389/fpsyt.2025.1539158 (PMC11891218; doi:10.3389/fpsyt.2025.1539158)
Supplement: Supplementary file 1 [file SupplementaryFile1.docx]

Supplementary Material

Supplementary Table 1. Detection methods for biochemical indicators

|  | Method | Kit | Instrument |
| --- | --- | --- | --- |
| ALB | BCG (Bromocresol Green) method | - | LABOSPECT 008AS automatic biochemistry analyzer (HITACHI) |
| TBIL | Diazo method | - |  |
| UA | Uricase-Peroxidase method | - |  |
| GSH | DTNB (Ellman's reagent) method | ADS-W-G001 | SMR60047 microplate reader (USCNK) |
| SOD | WST-8 (Water Soluble Tetrazolium-8) method | ADS-W-KY011 |  |
| GSH-Px | Enzyme catalysis method | A005-1 |  |
| MDA | TBA (Thiobarbituric acid) method | ADS-W-YH002 |  |
| NO | Nitrate reduction test | ADS-W-N005-96 |  |
| IL-6 | enzyme-linked immunosorbent assay (ELISA) | MM-0049H1 | Type 352 microplate reader (Labsystems Multiskan MS) |
| TNF-α |  | MM-0122H1 |  |
| CRP |  | MM-0135H1 |  |
| SII | SII = peripheral platelet count ×  neutrophil count / lymphocyte count | - | BC-6800 Plus Automated Hematology Analyzer (MINDRAY) |

Supplementary Table 2. Univariate logistic regression analysis for the association between the indicators with suicide attempt (SA) and suicide intent in adolescents having MDD

|  | Suicide attempt (SA) | |  | Suicide intent | |
| --- | --- | --- | --- | --- | --- |
|  | OR (95% CI) | *p*-value |  | OR (95% CI) | *p*-value |
| Age, years | 1.19 (0.95, 1.51) | 0.141 |  | 0.92 (0.73, 1.16) | 0.496 |
| Gender |  |  |  |  |  |
| male | ref. |  |  | ref. |  |
| female | 1.75 (0.70, 4.59) | 0.240 |  | 2.60 (1.04, 6.88) | 0.046^*^ |
| BMI | 1.01 (0.92, 1.12) | 0.760 |  | 1.01 (0.92, 1.11) | 0.895 |
| Course of MDD, month | 1.02 (0.99, 1.05) | 0.130 |  | 1.01 (0.98, 1.04) | 0.466 |
| Family history |  |  |  |  |  |
| no | ref. |  |  | ref. |  |
| yes | 1.36 (0.56, 3.34) | 0.501 |  | 1.12 (0.46, 2.78) | 0.810 |
| ALB, g/L | 0.94 (0.81, 1.09) | 0.435 |  | 0.89 (0.76, 1.03) | 0.112 |
| TBIL, umol/L | 0.94 (0.86, 1.02) | 0.152 |  | 0.89 (0.81, 0.97) | 0.014^*^ |
| UA, umol/L | 1.00 (0.99, 1.00) | 0.616 |  | 1.00 (0.99, 1.00) | 0.096 |
| GSH, umol/L | 1.00 (0.98, 1.03) | 0.807 |  | 1.00 (0.98, 1.03) | 0.854 |
| SOD, U/mL | 1.30 (1.11, 1.56) | 0.002^**^ |  | 1.34 (1.14, 1.61) | 0.001^***^ |
| GSH-Px, U/mL | 1.02 (0.99, 1.04) | 0.147 |  | 1.00 (0.98, 1.02) | 0.879 |
| MDA, nmol/mL | 1.00 (0.93, 1.07) | 0.949 |  | 0.96 (0.89, 1.04) | 0.304 |
| NO, μmol/L | 0.86 (0.74, 0.99) | 0.046^*^ |  | 0.96 (0.83, 1.10) | 0.537 |
| IL-6, ng/L | 0.88 (0.71, 1.08) | 0.229 |  | 0.79 (0.63, 0.97) | 0.029^*^ |
| TNF-α, ng/L | 1.01 (1.00, 1.02) | 0.078 |  | 1.01 (1.00, 1.02) | 0.036^*^ |
| CRP, μg/L | 1.00 (1.00, 1.00) | 0.652 |  | 1.00 (1.00, 1.00) | 0.726 |
| SII | 1.00 (1.00, 1.00) | 0.605 |  | 1.00 (1.00, 1.00) | 0.965 |
| NSSI |  |  |  |  |  |
| no | ref. |  |  | ref. |  |
| yes | 1.72 (0.74, 4.11) | 0.215 |  | 3.96 (1.66, 9.98) | 0.002^**^ |
| SAS | 1.07 (1.04, 1.11) | <0.001^***^ |  | 1.10 (1.06, 1.15) | <0.001^***^ |
| SDS | 1.06 (1.03, 1.10) | <0.001^***^ |  | 1.08 (1.05, 1.13) | <0.001^***^ |
| PSQI | 1.17 (1.07, 1.30) | 0.002^**^ |  | 1.18 (1.07, 1.32) | 0.001^**^ |
| ASLEC | 1.03 (1.01, 1.06) | 0.023^*^ |  | 1.03 (1.01, 1.06) | 0.020^*^ |
| IPPA |  |  |  |  |  |
| parent attachment | 0.95 (0.93, 0.98) | <0.001^***^ |  | 0.97 (0.95, 0.99) | 0.017^*^ |
| peer attachment | 1.00 (0.98, 1.02) | 0.956 |  | 1.00 (0.98, 1.02) | 0.974 |
| IRIDS | 1.06 (0.99, 1.14) | 0.090 |  | 1.14 (1.06, 1.23) | 0.001^**^ |
| TAS | 1.05 (1.01, 1.09) | 0.009^**^ |  | 1.05 (1.01, 1.09) | 0.009^**^ |
| SCSQ |  |  |  |  |  |
| negative coping pattern | ref. |  |  | ref. |  |
| positive coping pattern | 0.19 (0.06, 0.52) | 0.002^**^ |  | 0.35 (0.13, 0.87) | 0.027^*^ |
| BIS | 1.03 (1.00, 1.06) | 0.043^*^ |  | 1.01 (0.99, 1.04) | 0.364 |

* *P* < 0.05, ** *P* < 0.01, *** *P* < 0.001

Supplementary Table 3. The detailed information of datasets used in the MR analysis of the present study

| Trait | Ancestry | GWAS data source | Sample size | Reference |
| --- | --- | --- | --- | --- |
| Exposure |  |  |  |  |
| ALB | European | [ebi-a-GCST90025992](https://gwas.mrcieu.ac.uk/datasets/ebi-a-GCST90025992/) | 400,938 | Barton et al., 2021(1) |
| TBIL | European | [ebi-a-GCST90013872](https://gwas.mrcieu.ac.uk/datasets/ebi-a-GCST90013872/) | 388,303 | Mbatchou et al., 2021(2) |
| UA | European | [ebi-a-GCST90018977](https://gwas.mrcieu.ac.uk/datasets/ebi-a-GCST90018977/) | 343,836 | Sakaue et al., 2021(3) |
| GSH | African American or Afro-Caribbean (U.S.) | [GCST90176045](https://www.ebi.ac.uk/gwas/studies/GCST90176045) | 2,466 | Tahir et al., 2022(4) |
| SOD | European | [prot-a-2800](https://gwas.mrcieu.ac.uk/datasets/prot-a-2800/) | 3,301 | Sun et al., 2018(5) |
| GSH-Px | European | [prot-a-1265](https://gwas.mrcieu.ac.uk/datasets/prot-a-1265/) | 3,301 | Sun et al., 2018(5) |
| MDA | European (U.S.) | [GCST90257452](https://www.ebi.ac.uk/gwas/studies/GCST90257452) | 822 | Rhee et al., 2022(6) |
| CRP | European | [ebi-a-GCST90025959](https://gwas.mrcieu.ac.uk/datasets/ebi-a-GCST90025959/) | 436,939 | Barton et al., 2021(1) |
| IL-6 | European | [ebi-a-GCST90012005](https://gwas.mrcieu.ac.uk/datasets/ebi-a-GCST90012005/) | 21,758 | Folkersen et al., 2020(7) |
| TNF-α | European | [ebi-a-GCST004426](https://gwas.mrcieu.ac.uk/datasets/ebi-a-GCST004426/) | 3,454 | Ahola-Olli et al., 2017(8) |
| Outcome |  |  |  |  |
| Suicide attempt (SA) | European (U.K.) | [GCST90042799](https://www.ebi.ac.uk/gwas/studies/GCST90042799) | 6,183 | Jiang et al., 2021(9) |
|  | European | [finn-b-VWXY20_SUICI_OTHER_INTENTI_SELF_H](https://gwas.mrcieu.ac.uk/datasets/finn-b-VWXY20_SUICI_OTHER_INTENTI_SELF_H/) | 218,792 | NA |
| Suicidal ideation (SI) | European (U.K.) | [GCST90435861](https://www.ebi.ac.uk/gwas/studies/GCST90435861) | 365,819 | Zhou et al., 2018(10) |
| suicide behaviour | European (U.K.) | [GCST90435862](https://www.ebi.ac.uk/gwas/studies/GCST90435862) | 365,795 | Zhou et al., 2018(10) |

**Reference**

1. Barton AR, Sherman MA, Mukamel RE, Loh P-R. Whole-exome imputation within UK Biobank powers rare coding variant association and fine-mapping analyses. *Nat Genet* (2021) 53:1260–1269. doi: 10.1038/s41588-021-00892-1

2. Mbatchou J, Barnard L, Backman J, Marcketta A, Kosmicki JA, Ziyatdinov A, Benner C, O’Dushlaine C, Barber M, Boutkov B, et al. Computationally efficient whole-genome regression for quantitative and binary traits. *Nat Genet* (2021) 53:1097–1103. doi: 10.1038/s41588-021-00870-7

3. Sakaue S, Kanai M, Tanigawa Y, Karjalainen J, Kurki M, Koshiba S, Narita A, Konuma T, Yamamoto K, Akiyama M, et al. A cross-population atlas of genetic associations for 220 human phenotypes. *Nat Genet* (2021) 53:1415–1424. doi: 10.1038/s41588-021-00931-x

4. Tahir UA, Katz DH, Avila-Pachecho J, Bick AG, Pampana A, Robbins JM, Yu Z, Chen Z-Z, Benson MD, Cruz DE, et al. Whole Genome Association Study of the Plasma Metabolome Identifies Metabolites Linked to Cardiometabolic Disease in Black Individuals. *Nat Commun* (2022) 13:4923. doi: 10.1038/s41467-022-32275-3

5. Sun BB, Maranville JC, Peters JE, Stacey D, Staley JR, Blackshaw J, Burgess S, Jiang T, Paige E, Surendran P, et al. Genomic atlas of the human plasma proteome. *Nature* (2018) 558:73–79. doi: 10.1038/s41586-018-0175-2

6. Rhee EP, Surapaneni A, Zheng Z, Zhou L, Dutta D, Arking DE, Zhang J, Duong T, Chatterjee N, Luo S, et al. Trans-ethnic genome-wide association study of blood metabolites in the Chronic Renal Insufficiency Cohort (CRIC) study. *Kidney Int* (2022) 101:814–823. doi: 10.1016/j.kint.2022.01.014

7. Folkersen L, Gustafsson S, Wang Q, Hansen DH, Hedman ÅK, Schork A, Page K, Zhernakova DV, Wu Y, Peters J, et al. Genomic and drug target evaluation of 90 cardiovascular proteins in 30,931 individuals. *Nat Metab* (2020) 2:1135–1148. doi: 10.1038/s42255-020-00287-2

8. Ahola-Olli AV, Würtz P, Havulinna AS, Aalto K, Pitkänen N, Lehtimäki T, Kähönen M, Lyytikäinen L-P, Raitoharju E, Seppälä I, et al. Genome-wide Association Study Identifies 27 Loci Influencing Concentrations of Circulating Cytokines and Growth Factors. *Am J Hum Genet* (2017) 100:40–50. doi: 10.1016/j.ajhg.2016.11.007

9. Jiang L, Zheng Z, Fang H, Yang J. A generalized linear mixed model association tool for biobank-scale data. *Nat Genet* (2021) 53:1616–1621. doi: 10.1038/s41588-021-00954-4

10. Zhou W, Nielsen JB, Fritsche LG, Dey R, Gabrielsen ME, Wolford BN, LeFaive J, VandeHaar P, Gagliano SA, Gifford A, et al. Efficiently controlling for case-control imbalance and sample relatedness in large-scale genetic association studies. *Nat Genet* (2018) 50:1335–1341. doi: 10.1038/s41588-018-0184-y

Supplementary Table 4. The MR analysis results of the causal effects of serum indicator levels on suicide ideation (SI), suicide attempt (SA), and suicide behavior (SB).

| Exposure | Outcome | P_p_ | P_h_ | MR method | No. SNPs | OR (95% CI) | P-value |
| --- | --- | --- | --- | --- | --- | --- | --- |
| ALB | SA (UKB) | 0.887 | 0.534 | **Fixed-effect IVW** | **220** | **0.999 (0.791, 1.261)** | **0.992** |
|  |  |  |  | Random effect IVW | 220 | 0.999 (0.791, 1.261) | 0.992 |
|  |  |  |  | Maximum likelihood | 220 | 0.999 (0.790, 1.262) | 0.992 |
|  |  |  |  | MR Egger | 220 | 1.024 (0.672, 1.562) | 0.911 |
|  |  |  |  | Weighted median | 220 | 0.895 (0.609, 1.315) | 0.572 |
|  | SA (Finn) | 0.234 | **0.003** | Fixed-effect IVW | 224 | 1.002 (0.950, 1.057) | 0.941 |
|  |  |  |  | **Random effect IVW** | **224** | **1.002 (0.943, 1.065)** | **0.948** |
|  |  |  |  | Maximum likelihood | 224 | 1.002 (0.949, 1.058) | 0.941 |
|  |  |  |  | MR Egger | 224 | 1.063 (0.948, 1.192) | 0.296 |
|  |  |  |  | Weighted median | 224 | 0.984 (0.900, 1.076) | 0.727 |
|  | SI | 0.210 | 0.997 | **Fixed-effect IVW** | **221** | **0.787 (0.486, 1.275)** | **0.330** |
|  |  |  |  | Random effect IVW | 221 | 0.787 (0.486, 1.275) | 0.330 |
|  |  |  |  | Maximum likelihood | 221 | 0.786 (0.484, 1.276) | 0.331 |
|  |  |  |  | MR Egger | 221 | 0.491 (0.204, 1.183) | 0.114 |
|  |  |  |  | Weighted median | 221 | 0.630 (0.285, 1.396) | 0.255 |
|  | SB | 0.291 | 0.998 | **Fixed-effect IVW** | **221** | **0.841 (0.510, 1.386)** | **0.496** |
|  |  |  |  | Random effect IVW | 221 | 0.841 (0.510, 1.386) | 0.496 |
|  |  |  |  | Maximum likelihood | 221 | 0.841 (0.509, 1.390) | 0.500 |
|  |  |  |  | MR Egger | 221 | 0.557 (0.223, 1.386) | 0.209 |
|  |  |  |  | Weighted median | 221 | 0.783 (0.355, 1.728) | 0.545 |
| TBIL | SA (UKB) | 0.717 | 0.904 | **Fixed-effect IVW** | **165** | **0.992 (0.776, 1.267)** | **0.948** |
|  |  |  |  | Random effect IVW | 165 | 0.992 (0.776, 1.267) | 0.948 |
|  |  |  |  | Maximum likelihood | 165 | 0.992 (0.776, 1.268) | 0.948 |
|  |  |  |  | MR Egger | 165 | 1.035 (0.740, 1.447) | 0.841 |
|  |  |  |  | Weighted median | 165 | 0.879 (0.602, 1.284) | 0.504 |
|  | SA (Finn) | 0.880 | **<0.001** | Fixed-effect IVW | 172 | 1.017 (0.973, 1.063) | 0.450 |
|  |  |  |  | **Random effect IVW** | **172** | **1.017 (0.965, 1.072)** | **0.525** |
|  |  |  |  | Maximum likelihood | 172 | 1.017 (0.973, 1.063) | 0.450 |
|  |  |  |  | MR Egger | 172 | 1.014 (0.950, 1.082) | 0.671 |
|  |  |  |  | Weighted median | 172 | 1.041 (0.971, 1.116) | 0.262 |
|  | SI | 0.090 | 0.821 | **Fixed-effect IVW** | **158** | **1.449 (0.880, 2.385)** | **0.145** |
|  |  |  |  | Random effect IVW | 158 | 1.449 (0.880, 2.385) | 0.145 |
|  |  |  |  | Maximum likelihood | 158 | 1.449 (0.879, 2.389) | 0.146 |
|  |  |  |  | MR Egger | 158 | 0.991 (0.511, 1.923) | 0.980 |
|  |  |  |  | Weighted median | 158 | 1.367 (0.649, 2.876) | 0.411 |
|  | SB | 0.146 | 0.960 | **Fixed-effect IVW** | **158** | **1.434 (0.855, 2.403)** | **0.172** |
|  |  |  |  | Random effect IVW | 158 | 1.434 (0.855, 2.403) | 0.172 |
|  |  |  |  | Maximum likelihood | 158 | 1.434 (0.854, 2.406) | 0.173 |
|  |  |  |  | MR Egger | 158 | 1.025 (0.516, 2.034) | 0.945 |
|  |  |  |  | Weighted median | 158 | 1.434 (0.650, 3.166) | 0.372 |
| UA | SA (UKB) | 0.721 | 0.210 | **Fixed-effect IVW** | **240** | **0.802 (0.665, 0.968)** | **0.021** |
|  |  |  |  | Random effect IVW | 240 | 0.802 (0.661, 0.975) | 0.026 |
|  |  |  |  | Maximum likelihood | 240 | 0.802 (0.665, 0.968) | 0.022 |
|  |  |  |  | MR Egger | 240 | 0.778 (0.600, 1.008) | 0.058 |
|  |  |  |  | Weighted median | 240 | 0.855 (0.654, 1.119) | 0.255 |
|  | SA (Finn) | 0.481 | **<0.001** | Fixed-effect IVW | 248 | 0.953 (0.914, 0.994) | 0.026 |
|  |  |  |  | **Random effect IVW** | **248** | **0.953 (0.905, 1.004)** | **0.070** |
|  |  |  |  | Maximum likelihood | 248 | 0.953 (0.913, 0.994) | 0.027 |
|  |  |  |  | MR Egger | 248 | 0.937 (0.874, 1.005) | 0.070 |
|  |  |  |  | Weighted median | 248 | 0.940 (0.884, 0.999) | 0.046 |
|  | SI | 0.682 | 0.448 | **Fixed-effect IVW** | **226** | **0.634 (0.365, 1.101)** | **0.105** |
|  |  |  |  | Random effect IVW | 226 | 0.634 (0.365, 1.103) | 0.107 |
|  |  |  |  | Maximum likelihood | 226 | 0.633 (0.364, 1.102) | 0.106 |
|  |  |  |  | MR Egger | 226 | 0.766 (0.266, 2.204) | 0.621 |
|  |  |  |  | Weighted median | 226 | 0.671 (0.221, 2.037) | 0.481 |
|  | SB | 0.897 | 0.454 | **Fixed-effect IVW** | **226** | **0.636 (0.359, 1.125)** | **0.120** |
|  |  |  |  | Random effect IVW | 226 | 0.636 (0.358, 1.128) | 0.121 |
|  |  |  |  | Maximum likelihood | 226 | 0.636 (0.358, 1.129) | 0.122 |
|  |  |  |  | MR Egger | 226 | 0.676 (0.226, 2.020) | 0.484 |
|  |  |  |  | Weighted median | 226 | 0.850 (0.286, 2.527) | 0.771 |
| GSH* | SA (UKB) | 0.998 | 0.744 | **Fixed-effect IVW** | **12** | **1.019 (0.906, 1.145)** | **0.758** |
|  |  |  |  | Random effect IVW | 12 | 1.019 (0.906, 1.145) | 0.758 |
|  |  |  |  | Maximum likelihood | 12 | 1.019 (0.906, 1.146) | 0.757 |
|  |  |  |  | MR Egger | 12 | 1.018 (0.831, 1.248) | 0.865 |
|  |  |  |  | Weighted median | 12 | 1.029 (0.876, 1.209) | 0.730 |
|  | SA (Finn) | 0.240 | 0.792 | **Fixed-effect IVW** | **11** | **0.988 (0.960, 1.017)** | **0.405** |
|  |  |  |  | Random effect IVW | 11 | 0.988 (0.960, 1.017) | 0.405 |
|  |  |  |  | Maximum likelihood | 11 | 0.988 (0.959, 1.017) | 0.399 |
|  |  |  |  | MR Egger | 11 | 0.958 (0.907, 1.013) | 0.166 |
|  |  |  |  | Weighted median | 11 | 0.984 (0.946, 1.023) | 0.409 |
|  | SI | 0.528 | 0.076 | **Fixed-effect IVW** | **11** | **0.993 (0.786, 1.255)** | **0.955** |
|  |  |  |  | Random effect IVW | 11 | 0.993 (0.732, 1.347) | 0.966 |
|  |  |  |  | Maximum likelihood | 11 | 0.993 (0.782, 1.261) | 0.955 |
|  |  |  |  | MR Egger | 11 | 1.145 (0.676, 1.939) | 0.627 |
|  |  |  |  | Weighted median | 11 | 1.043 (0.757, 1.437) | 0.796 |
|  | SB | 0.495 | 0.069 | **Fixed-effect IVW** | **11** | **0.992 (0.779, 1.264)** | **0.950** |
|  |  |  |  | Random effect IVW | 11 | 0.992 (0.723, 1.363) | 0.962 |
|  |  |  |  | Maximum likelihood | 11 | 0.992 (0.776, 1.269) | 0.951 |
|  |  |  |  | MR Egger | 11 | 1.162 (0.674, 2.003) | 0.601 |
|  |  |  |  | Weighted median | 11 | 1.037 (0.750, 1.434) | 0.825 |
| SOD* | SA (UKB) | 0.813 | 0.259 | **Fixed-effect IVW** | **12** | **1.095 (0.925, 1.295)** | **0.292** |
|  |  |  |  | Random effect IVW | 12 | 1.095 (0.908, 1.319) | 0.342 |
|  |  |  |  | Maximum likelihood | 12 | 1.099 (0.925, 1.305) | 0.282 |
|  |  |  |  | MR Egger | 12 | 1.147 (0.752, 1.749) | 0.540 |
|  |  |  |  | Weighted median | 12 | 1.121 (0.885, 1.420) | 0.342 |
|  | SA (Finn) | 0.676 | 0.248 | **Fixed-effect IVW** | **13** | **0.980 (0.946, 1.014)** | **0.246** |
|  |  |  |  | Random effect IVW | 13 | 0.980 (0.943, 1.018) | 0.297 |
|  |  |  |  | Maximum likelihood | 13 | 0.979 (0.944, 1.015) | 0.244 |
|  |  |  |  | MR Egger | 13 | 0.963 (0.884, 1.050) | 0.415 |
|  |  |  |  | Weighted median | 13 | 0.953 (0.907, 1.002) | 0.059 |
|  | SI | 0.866 | 0.302 | **Fixed-effect IVW** | **12** | **1.249 (0.881, 1.771)** | **0.212** |
|  |  |  |  | Random effect IVW | 12 | 1.249 (0.856, 1.822) | 0.249 |
|  |  |  |  | Maximum likelihood | 12 | 1.264 (0.884, 1.809) | 0.199 |
|  |  |  |  | MR Egger | 12 | 1.336 (0.565, 3.162) | 0.524 |
|  |  |  |  | Weighted median | 12 | 1.037 (0.638, 1.686) | 0.882 |
|  | SB | 0.659 | 0.270 | **Fixed-effect IVW** | **12** | **1.195 (0.832, 1.717)** | **0.335** |
|  |  |  |  | Random effect IVW | 12 | 1.195 (0.802, 1.781) | 0.381 |
|  |  |  |  | Maximum likelihood | 12 | 1.205 (0.832, 1.746) | 0.324 |
|  |  |  |  | MR Egger | 12 | 1.440 (0.584, 3.549) | 0.447 |
|  |  |  |  | Weighted median | 12 | 1.058 (0.636, 1.761) | 0.827 |
| GSH-Px* | SA (UKB) | 0.136 | 0.676 | **Fixed-effect IVW** | **13** | **0.974 (0.861, 1.100)** | **0.668** |
|  |  |  |  | Random effect IVW | 13 | 0.974 (0.861, 1.100) | 0.668 |
|  |  |  |  | Maximum likelihood | 13 | 0.973 (0.860, 1.101) | 0.665 |
|  |  |  |  | MR Egger | 13 | 1.125 (0.908, 1.393) | 0.305 |
|  |  |  |  | Weighted median | 13 | 0.987 (0.847, 1.151) | 0.871 |
|  | SA (Finn) | 0.912 | 0.906 | **Fixed-effect IVW** | **14** | **0.981 (0.954, 1.009)** | **0.187** |
|  |  |  |  | Random effect IVW | 14 | 0.981 (0.954, 1.009) | 0.187 |
|  |  |  |  | Maximum likelihood | 14 | 0.981 (0.954, 1.009) | 0.184 |
|  |  |  |  | MR Egger | 14 | 0.979 (0.930, 1.030) | 0.429 |
|  |  |  |  | Weighted median | 14 | 0.976 (0.941, 1.013) | 0.200 |
|  | SI | 0.432 | 0.259 | **Fixed-effect IVW** | **13** | **0.873 (0.678, 1.124)** | **0.292** |
|  |  |  |  | Random effect IVW | 13 | 0.873 (0.661, 1.154) | 0.341 |
|  |  |  |  | Maximum likelihood | 13 | 0.870 (0.674, 1.124) | 0.288 |
|  |  |  |  | MR Egger | 13 | 0.733 (0.441, 1.218) | 0.255 |
|  |  |  |  | Weighted median | 13 | 0.805 (0.582, 1.114) | 0.191 |
|  | SB | 0.544 | 0.368 | **Fixed-effect IVW** | **13** | **0.850 (0.654, 1.105)** | **0.224** |
|  |  |  |  | Random effect IVW | 13 | 0.850 (0.647, 1.117) | 0.243 |
|  |  |  |  | Maximum likelihood | 13 | 0.848 (0.651, 1.106) | 0.224 |
|  |  |  |  | MR Egger | 13 | 0.744 (0.450, 1.230) | 0.273 |
|  |  |  |  | Weighted median | 13 | 0.815 (0.580, 1.144) | 0.237 |
| MDA* | SA (UKB) | 0.941 | 0.517 | **Fixed-effect IVW** | **12** | **0.999 (0.858, 1.164)** | **0.994** |
|  |  |  |  | Random effect IVW | 12 | 0.999 (0.858, 1.164) | 0.994 |
|  |  |  |  | Maximum likelihood | 12 | 0.999 (0.855, 1.168) | 0.994 |
|  |  |  |  | MR Egger | 12 | 1.014 (0.675, 1.524) | 0.947 |
|  |  |  |  | Weighted median | 12 | 0.993 (0.802, 1.229) | 0.949 |
|  | SA (Finn) | 0.131 | 0.391 | **Fixed-effect IVW** | **12** | **1.024 (0.989, 1.060)** | **0.186** |
|  |  |  |  | Random effect IVW | 12 | 1.024 (0.988, 1.061) | 0.198 |
|  |  |  |  | Maximum likelihood | 12 | 1.024 (0.989, 1.061) | 0.186 |
|  |  |  |  | MR Egger | 12 | 1.092 (1.004, 1.189) | 0.068 |
|  |  |  |  | Weighted median | 12 | 1.059 (1.009, 1.112) | 0.021 |
|  | SI | 0.454 | 0.711 | **Fixed-effect IVW** | **11** | **0.740 (0.530, 1.032)** | **0.076** |
|  |  |  |  | Random effect IVW | 11 | 0.740 (0.530, 1.032) | 0.076 |
|  |  |  |  | Maximum likelihood | 11 | 0.744 (0.529, 1.047) | 0.090 |
|  |  |  |  | MR Egger | 11 | 1.051 (0.411, 2.691) | 0.919 |
|  |  |  |  | Weighted median | 11 | 0.693 (0.440, 1.091) | 0.113 |
|  | SB | 0.362 | 0.702 | **Fixed-effect IVW** | **11** | **0.734 (0.520, 1.036)** | **0.078** |
|  |  |  |  | Random effect IVW | 11 | 0.734 (0.520, 1.036) | 0.078 |
|  |  |  |  | Maximum likelihood | 11 | 0.728 (0.510, 1.038) | 0.079 |
|  |  |  |  | MR Egger | 11 | 1.145 (0.433, 3.027) | 0.791 |
|  |  |  |  | Weighted median | 11 | 0.689 (0.433, 1.097) | 0.116 |
| CRP | SA (UKB) | 0.489 | 0.313 | **Fixed-effect IVW** | **246** | **1.040 (0.893, 1.211)** | **0.615** |
|  |  |  |  | Random effect IVW | 246 | 1.040 (0.890, 1.215) | 0.622 |
|  |  |  |  | Maximum likelihood | 246 | 1.040 (0.893, 1.212) | 0.612 |
|  |  |  |  | MR Egger | 246 | 0.992 (0.807, 1.218) | 0.937 |
|  |  |  |  | Weighted median | 246 | 0.962 (0.762, 1.213) | 0.742 |
|  | SA (Finn) | 0.249 | **0.005** | Fixed-effect IVW | 248 | 0.990 (0.954, 1.028) | 0.610 |
|  |  |  |  | **Random effect IVW** | **248** | **0.990 (0.950, 1.033)** | **0.648** |
|  |  |  |  | Maximum likelihood | 248 | 0.990 (0.954, 1.028) | 0.613 |
|  |  |  |  | MR Egger | 248 | 1.014 (0.957, 1.074) | 0.640 |
|  |  |  |  | Weighted median | 248 | 1.021 (0.958, 1.088) | 0.517 |
|  | SI | 0.479 | 0.967 | **Fixed-effect IVW** | **246** | **1.126 (0.821, 1.544)** | **0.463** |
|  |  |  |  | Random effect IVW | 246 | 1.126 (0.821, 1.544) | 0.463 |
|  |  |  |  | Maximum likelihood | 246 | 1.126 (0.820, 1.545) | 0.464 |
|  |  |  |  | MR Egger | 246 | 1.242 (0.819, 1.884) | 0.309 |
|  |  |  |  | Weighted median | 246 | 1.082 (0.659, 1.777) | 0.755 |
|  | SB | 0.314 | 0.961 | **Fixed-effect IVW** | **246** | **1.200 (0.865, 1.666)** | **0.275** |
|  |  |  |  | Random effect IVW | 246 | 1.200 (0.865, 1.666) | 0.275 |
|  |  |  |  | Maximum likelihood | 246 | 1.200 (0.864, 1.666) | 0.277 |
|  |  |  |  | MR Egger | 246 | 1.387 (0.901, 2.137) | 0.139 |
|  |  |  |  | Weighted median | 246 | 1.328 (0.802, 2.199) | 0.270 |
| IL-6* | SA (UKB) | 0.524 | 0.819 | **Fixed-effect IVW** | **14** | **0.770 (0.599, 0.989)** | **0.040** |
|  |  |  |  | Random effect IVW | 14 | 0.770 (0.599, 0.989) | 0.040 |
|  |  |  |  | Maximum likelihood | 14 | 0.774 (0.600, 0.997) | 0.048 |
|  |  |  |  | MR Egger | 14 | 0.670 (0.414, 1.086) | 0.130 |
|  |  |  |  | Weighted median | 14 | 0.645 (0.456, 0.912) | 0.013 |
|  | SA (Finn) | 0.201 | 0.085 | **Fixed-effect IVW** | **12** | **0.955 (0.899, 1.013)** | **0.127** |
|  |  |  |  | Random effect IVW | 12 | 0.955 (0.884, 1.030) | 0.231 |
|  |  |  |  | Maximum likelihood | 12 | 0.953 (0.897, 1.014) | 0.127 |
|  |  |  |  | MR Egger | 12 | 1.043 (0.901, 1.208) | 0.586 |
|  |  |  |  | Weighted median | 12 | 0.994 (0.913, 1.081) | 0.884 |
|  | SI | 0.727 | 0.981 | **Fixed-effect IVW** | **14** | **1.132 (0.674, 1.902)** | **0.639** |
|  |  |  |  | Random effect IVW | 14 | 1.132 (0.674, 1.902) | 0.639 |
|  |  |  |  | Maximum likelihood | 14 | 1.133 (0.672, 1.908) | 0.640 |
|  |  |  |  | MR Egger | 14 | 0.970 (0.359, 2.622) | 0.953 |
|  |  |  |  | Weighted median | 14 | 1.264 (0.617, 2.590) | 0.522 |
|  | SB | 0.707 | 0.988 | **Fixed-effect IVW** | **14** | **1.167 (0.681, 1.999)** | **0.574** |
|  |  |  |  | Random effect IVW | 14 | 1.167 (0.681, 1.999) | 0.574 |
|  |  |  |  | Maximum likelihood | 14 | 1.170 (0.682, 2.010) | 0.569 |
|  |  |  |  | MR Egger | 14 | 0.982 (0.350, 2.754) | 0.972 |
|  |  |  |  | Weighted median | 14 | 1.206 (0.575, 2.527) | 0.620 |
| TNF-α* | SA (UKB) | 0.684 | 0.527 | **Fixed-effect IVW** | **5** | **1.202 (0.970, 1.488)** | **0.092** |
|  |  |  |  | Random effect IVW | 5 | 1.202 (0.970, 1.488) | 0.092 |
|  |  |  |  | Maximum likelihood | 5 | 1.206 (0.971, 1.498) | 0.091 |
|  |  |  |  | MR Egger | 5 | 1.273 (0.915, 1.772) | 0.248 |
|  |  |  |  | Weighted median | 5 | 1.256 (0.971, 1.624) | 0.083 |
|  | SA (Finn) | 0.224 | 0.068 | **Fixed-effect IVW** | **5** | **1.012 (0.954, 1.074)** | **0.692** |
|  |  |  |  | Random effect IVW | 5 | 1.012 (0.927, 1.104) | 0.789 |
|  |  |  |  | Maximum likelihood | 5 | 1.013 (0.953, 1.077) | 0.681 |
|  |  |  |  | MR Egger | 5 | 1.096 (0.965, 1.245) | 0.252 |
|  |  |  |  | Weighted median | 5 | 0.990 (0.908, 1.080) | 0.820 |
|  | SI | 0.237 | 0.485 | **Fixed-effect IVW** | **5** | **0.688 (0.442, 1.072)** | **0.098** |
|  |  |  |  | Random effect IVW | 5 | 0.688 (0.442, 1.072) | 0.098 |
|  |  |  |  | Maximum likelihood | 5 | 0.679 (0.426, 1.080) | 0.102 |
|  |  |  |  | MR Egger | 5 | 1.018 (0.514, 2.017) | 0.963 |
|  |  |  |  | Weighted median | 5 | 0.776 (0.456, 1.320) | 0.349 |
|  | SB | 0.230 | 0.567 | **Fixed-effect IVW** | **5** | **0.674 (0.426, 1.066)** | **0.092** |
|  |  |  |  | Random effect IVW | 5 | 0.674 (0.426, 1.066) | 0.092 |
|  |  |  |  | Maximum likelihood | 5 | 0.666 (0.413, 1.075) | 0.096 |
|  |  |  |  | MR Egger | 5 | 1.016 (0.502, 2.057) | 0.967 |
|  |  |  |  | Weighted median | 5 | 0.812 (0.449, 1.467) | 0.490 |

* A less stringent cut-off value of P < 5×10^-6^ was used in the screening of instrumental variables.

The best causal estimation highlighted in bold.

P_p_, P-value for pleiotropy; P_h_, P-value for heterogeneity;


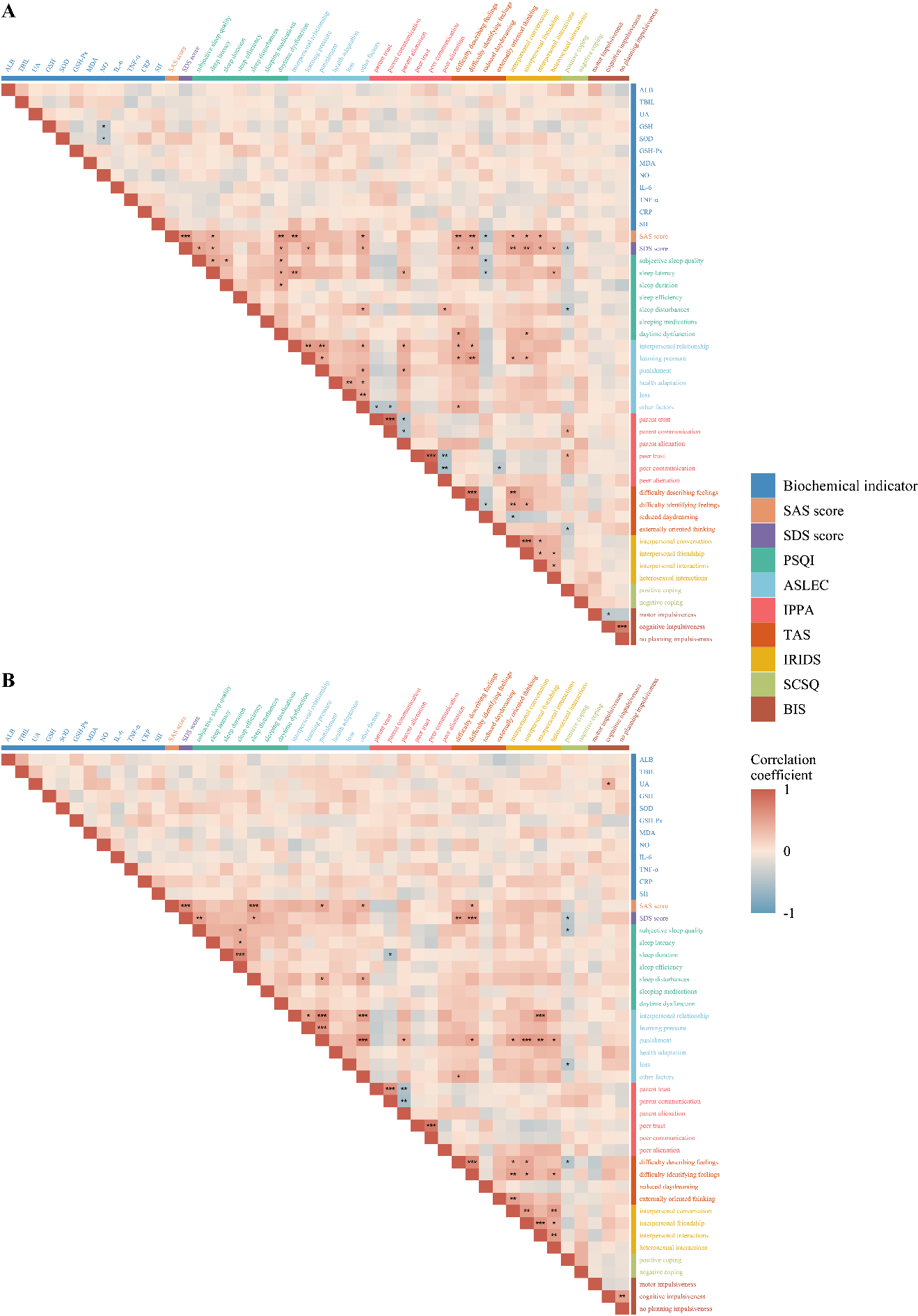


**Supplementary Figure 1.** Heatmaps presenting the Spearman correlation coefficients between all indicators in the MDD (A) and MDD+SA (B) groups among the participants with adolescent MDD. * Adjusted P < 0.05, ** Adjusted P < 0.01, and *** Adjusted P < 0.001.


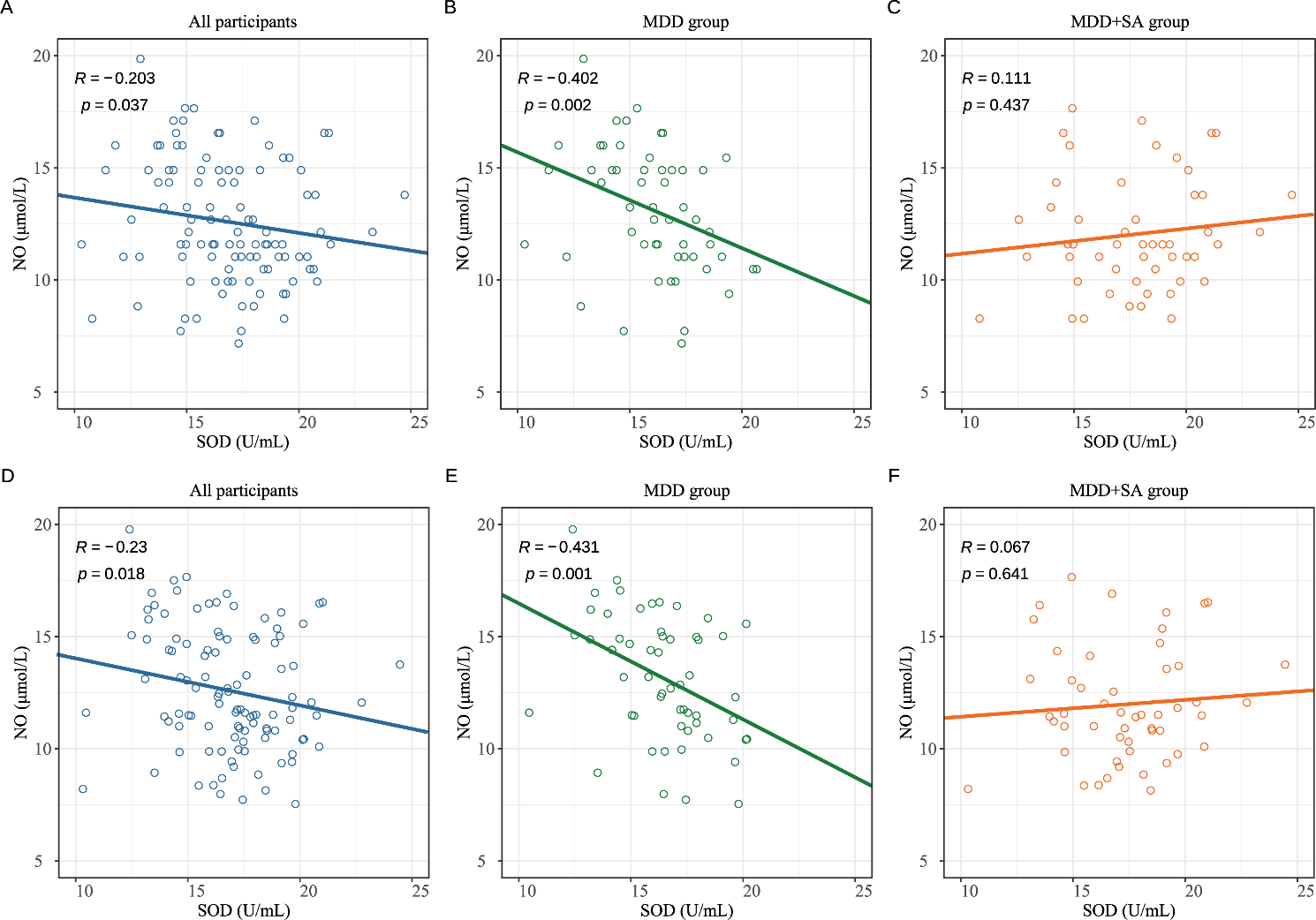


Supplementary Figure 2. Scatter plots A-C depict the correlations between the serum SOD activity and NO levels in all participants (A), MDD (B), and MDD+SA group (C). Scatter plots D-F show the results of the partial correlation analysis after controlling for SDS scores, which assess the severity of depressive symptoms. R and p represent the Spearman correlation coefficient and the corresponding p-value.


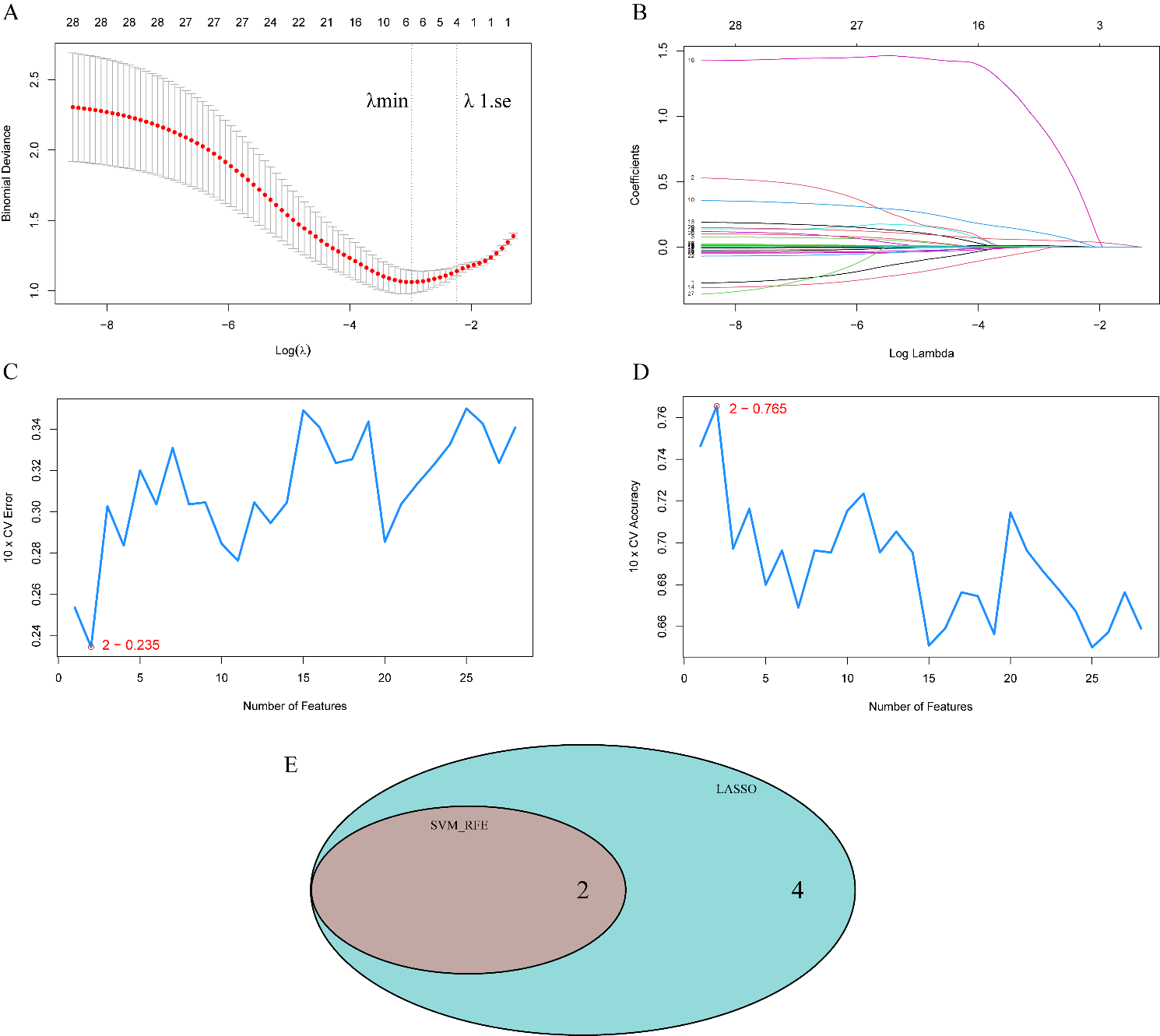


Supplementary Figure 3. Selection of risk variables using the least absolute shrinkage and selection operator (LASSO) and support vector machine-recursive feature elimination (SVM-RFE) model. (A) The variation characteristics of the coefficient of variables; (B) the selection process of the optimum value of the parameter λ in the LASSO regression model by cross-validation method; (C, D) SVM–RFE model identified 21 candidates with an error of 0.235 and an accuracy of 0.765; (E) Venn plot shows the overlapped candidates.
